# Negative Short-Term Outcome of Detoxification Therapy in Chronic Migraine With Medication Overuse Headache: Role for Early Life Traumatic Experiences and Recent Stressful Events

**DOI:** 10.3389/fneur.2019.00173

**Published:** 2019-03-07

**Authors:** Sara Bottiroli, Federica Galli, Michele Viana, Roberto De Icco, Vito Bitetto, Marta Allena, Stefania Pazzi, Grazia Sances, Cristina Tassorelli

**Affiliations:** ^1^Faculty of Law, Giustino Fortunato University, Benevento, Italy; ^2^Headache Science Centre, IRCCS Mondino Foundation, Pavia, Italy; ^3^Department of Health Sciences, University of Milan, Milan, Italy; ^4^Headache Group, Department of Basic and Clinical Neurosciences, King's College London, London, United Kingdom; ^5^Headache Center, Institute of the Neurocenter of Southern Switzerland (NSI), Regional Hospital Lugano, Lugano, Switzerland; ^6^Department of Brain and Behavioral Sciences, University of Pavia, Pavia, Italy

**Keywords:** medication-overuse headache, follow-up, psychological factors, childhood traumas, stressful events

## Abstract

**Background:** Early traumatic experiences and Stressful episodes appear to be associated to the development and perpetuation of chronic pain disorders and to dependence-related behaviors.

**Objective:** The present study evaluated whether these factors can be predictors, together with psychiatric conditions, of the outcome of a detoxification treatment in patients suffering from chronic migraine and medication-overuse headache in a 2-month follow-up.

**Methods:** Consecutive patients undergoing a detoxification program as therapy for treating chronic migraine and medication overuse headache at the Pavia Headache Center were analyzed. During this program, lasting about 1 week, all patients received the standard CARE in-patient withdrawal protocol, which consisted in discontinuing abruptly the overused drug(s) and receiving daily detoxification therapy. Data on childhood traumatic events and recent stressful ones were analyzed by means of the Childhood Trauma Questionnaire and Stressful life-events Questionnaire. Psychiatric conditions were evaluated using the Structured Clinical Interview for Diagnostic and Statistical Manual of mental disorders.

**Results:** A total of 166 (80% females; mean age 44.7) patients completed the follow-up at 2 months after the detoxification program: of these 118 (71%) (78% females; mean age 44.7) stopped overuse and reverted to an episodic pattern of headache (Group A); 19 (11%) (89% females; mean age 41.3) kept overusing and maintained a chronic pattern of headache (Group B); and 29 (18%) (79% females; mean age 46.9) stopped overuse without any benefit on headache frequency (Group C). At the multivariate analyses, a higher number of early life emotional distress (Odds Ratio 11.096; *p* = 0.037) arose as a prognostic factor for the outcome in Group B, while major depression during life-time (Odds Ratio 3.703; *p* = 0.006) and higher number of severe stressful episodes in the past 10 years (Odds Ratio 1.679; *p* = 0.045) were prognostic factors for the outcome of Group C.

**Conclusions:** Data suggest that early life traumas and stressful events have a negative impact on the outcome of the detoxification program in subjects overusing acute medication for headache. The history of emotional childhood traumas is associated to the failure to cease overuse, whereas recent very serious life events are associated to the persistence of headache chronicity.

## Introduction

Among the most popular and disabling neurological disorders, migraine is at the top of the list. In most sufferers, attacks recur episodically, even if in a small—but significant—portion of migraineurs the disease evolves into a chronic pattern, that is, chronic migraine (CM). Transition from episodic to CM often occurs in association with a progressive increase in the intake of acute medications, so that the large majority of patients with CM also fulfill criteria for Medication Overuse Headache (MOH) (1–3). MOH has a prevalence of 1–2% in the general population and a high economic burden on the society (4, 5). MOH is strongly associated with major depression, anxiety, social phobia, panic disorder, and personality disorders (6, 7). Besides the well-known overlap between psychiatric comorbidities and MOH, additional psychological factors are likely to play a role. For instance, studies in other types of chronic pain have shown the existence of an association between childhood traumatic episodes, stressful events, and the development and perpetuation of chronic pain conditions (8). This given the existence, in line with the bio-psychosocial model, of a composite interaction between psychological, psychosocial, and biological aspects reciprocally influencing each other (9). As for CM, we know that childhood traumas, such as physical and emotional abuse and neglect (10–12), and the experience of stressful events, such as health problems, financial troubles or migration (13–15), are capable to increase headache-related factors, such as frequency, severity, and chronicity. However, there are really few papers that investigated this topic specifically in MOH as a separate group among chronic headache sub-types. A study realized in Turkey (16) highlighted a similar pattern in term of early life maltreatments among MOH and chronic migraineurs. By contrast, our group lately discovered that early traumas in conjunction with history of depression, were predictors of MOH (17). As for current stressful events, a study by the Danish group (18) reported a strong link between MOH and stress, even if another study failed to identify such a relationship (19). Hence, there is a deep need to shed light on this topic.

Withdrawal from overused drug is generally regarded as the main step in the treatment of MOH (20, 21), which is associated to the improvement of headache in a large percentage of MOH sufferers (20, 22). According to the literature (1–3), withdrawal from overuse drugs is effective if the patient ceases overusing drugs for at least 2 months and the headache pattern reverts to episodic. In this case, it is likely to accurately identify the primary headache type according to existing classification criteria (1–3). A considerable portion of MOH patients does not benefit from overused drug withdrawal (23). Published studies are consistent in identifying two subgroups of non-responders: those who fail to stop drug overuse and those who manage to discontinue overused drug but do not experience any improvement of headache. A substantial body of literature, including our papers, has associated the failure to benefit from a detoxification programme to the presence of psychiatric comorbidity (24–26), but the directionality of the causal relationship is not clear because of the lack of long-term prospective studies. Similarly lacking is the data on the role of early traumas and current life events as determinant of outcome of CM with MOH. The investigation of these aspects could contribute to deepen our understanding of the disease and possibly allow to predict the patients who are more likely to respond to detoxification, according to their previous and current emotional history.

Keeping this in mind, this work is aimed at evaluating the possible role of childhood traumatic experiences and recent life events in the outcome of a detoxification treatment in patients suffering from chronic migraine and medication-overuse headache. The objective of this study is therefore to evaluate whether early traumatic experiences and stressful episodes can be predictors, together with psychiatric conditions such as Axis I disorders and affective state, of the outcome of a detoxification treatment in a 2-month follow-up. Our hypothesis is that early life traumatic experiences and stressful episodes could have a negative impact on detoxification outcome.

## Materials and Methods

### Sample Recruitment and Procedure

This study was conducted at the Headache Center (a tertiary referral center) of the C. Mondino National Neurological Institute in Pavia, Italy. All running patients with CM and MOH undergoing an inpatient detoxification program between April 2014 and May 2016 were enrolled. The ICDH-III-beta criteria (3) were used to make the diagnosis of MOH. The primary headache diagnosis was migraine for all patients and all of them satisfied criteria for chronic migraine. After completing the detoxification program, subjects were discharged from the hospital and scheduled for a follow-up at 2-months. We selected the 2-month follow-up in agreement with previous studies (27–29), as it represented the most conservative modality to evaluate the effect of the detoxification procedure itself, while minimizing the effect of spontaneous or prophylaxis-induced fluctuations. The local Ethics Committee approved this protocol and each single patient signed an informed consent before being enrolled in the study.

### Detoxification Program

During the hospitalization, which lasted about 1 week, all patients received the standard CARE in-patient withdrawal protocol described in detail elsewhere (22, 30). Briefly, the patients discontinued suddenly the overused drug(s) and received daily i.v. detoxification therapy: a saline solution 250 ml, delorazepam 0.25 mg (gradually eliminated over a period of 3–4 days), cyanocobalamine 2,500 mg, folic acid 0.70 mg, nicotinamide 12 mg, ascorbic acid 150 mg, glutathione 600 mg, metoclopramide 5 mg (stopped after 2 days), b.i.d. exception to the abrupt discontinuation of overused drugs was applied in case of butalbital overuse, when tapering doses of phenobarbital were added. If the patient was overusing narcotics, transdermal clonidine was added. For a short period of detoxification, we gave low-dose benzodiazepine to the patients. On day 4 or 5, we started with a preventive treatment, which was personalized in case of comorbidities and previous prophylactic therapy used by the patient. During hospitalization, only if patients suffered from terrible rebound headache, it was allowed to use rescue medication (100 mg of intramuscular ketoprofen). Upon discharge, it was prescribed a symptomatic drug of a different category from the one previously overused, to be used only for severe attacks and for no more than 5 days a month.

During hospitalization, all the patients received counseling regarding the risks associated to acute drug overuse and the best modalities to treat their attacks with acute drugs.

### Psychological Measures

Each patient was assessed by a neurologist, who used an *ad hoc* patient record form, concerning a complete personal and family history, with particular attention to the patient's headache history and profile of drug use. At the beginning of the study, patients were examined by a clinical psychologist by means of the Structured Clinical Interview for DSM-IV Disorders (SCID-I) (31) according to Axis I of the DSM-IV (32).

Participants also filled a series of self-report questionnaires. The Hospital Anxiety and Depression Scale (HADS) were used for evaluating anxious and depressive symptomatology (33) at admission. This questionnaire comprises seven items concerning depression and seven items for anxiety, graded on a four-point (0–3) Likert scale, so that possible scores ranged from 0 to 21 for both depression and anxiety. The Italian validation (34) showed that the 14-item scale had a high internal consistency (Cronbach's alpha 0.89 and 0.88) and a high discriminating power for all the psychiatric disorders (AUC = 0.89; 95% CI = 0.83–0.94).

Quality of life was assessed via a subtest of questions taken from the World Health Organization Quality of Life-BREF (WHOQoL) (35), comprising eight items to be answered on a five-point (1–5) Likert scale. Items concerned general quality of life, health status, psychological status, social relationships and environment. Higher scores in the WHOQoL indicate higher levels of quality of life. The Italian validation (36) of this instrument showed a good internal consistency, ranging from 0.65 for the social relationships domain to 0.80 for the physical domain. Test-retest reliability values (Spearman's correlation) were also good, ranging from 0.76 for the environment domain to 0.93 for the psychological domain.

Headache-related disability was assessed via the Migraine Disability Assessment (MIDAS) (37), formed by five questions investigating the impact of headache on everyday life activities (in terms of number of days affected) and two questions concerning headache frequency and intensity. Higher scores in the MIDAS indicate higher disability. The Italian validation (38) of the MIDAS demonstrated a good test-retest reliability (Spearman's correlation 0.77) and a good internal consistency (Cronbach's alpha 0.7).

For the assessment of maltreatment histories in early life, we adopted a shorter version of the Childhood Trauma Questionnaire (39) including 13 items assessing several kinds of childhood traumas such as emotional abuse, emotional neglect, physical abuse, physical neglect, and sexual abuse. An explanation of changes made in comparison to the original version of this questionnaire are explained in a previous paper from our group (40), in which we also preliminarily tested this questionnaire in a small group of CM+MOH subjects with known childhood trauma. In that study (40), this questionnaire proved as effective as the longer version in capturing the traumatic events, while requiring a shorter time for the compilation. Patients were requested to indicate whether they experienced one or more kinds of trauma. In the case of sexual abuse, we only considered presence/absence, while for physical and emotional traumas, we also asked the patients to indicate the number of experiences. The total score was calculated as the total number of traumatic experiences and, separately, the number of experiences for physical and emotional traumas.

Stressful life-events were assessed via a questionnaire–derived from Paykel et al. (41)—consisting in a list of 58 stressful life events (e.g., divorce, new work, moving, dismissal, etc). Patients were requested to tick those events that had occurred to them in the last 10 years; the total score was made by counting every stressful life episode signed by the patients. For each event, participants were also requested to rate how stressful the event was for them on a 5-points Likert scale ranging from mild to very serious.

### Definition of Outcomes

Detoxification was considered as successful when the patient stopped overuse of symptomatic medication and headache frequency was lower than 15 days per month during the follow-up period of 2 months. On the other hand, detoxification was defined as ineffective when (1) the patient failed to stop overuse during the follow-up or (2) overuse was stopped during the follow-up but headache kept a chronic pattern.

Therefore, considering the 2-month outcome, we subdivided patients according to the MOH diagnosis:

Group A: Detoxification was effective in interrupting overuse and in reverting the headache pattern to episodic during the 2-month follow-up period. This group was diagnosed with “definite MOH,” and patients were considered as “responders.”

Group B: Patients failed to cease drug overuse and headache maintained a chronic pattern in the 2-month follow-up period. This group was diagnosed with “probable MOH.”

Group C: Patients ceased drug overuse but their headache still remained chronic (≥15 days/month) during the 2-month observation period. For this group, MOH diagnosis was ruled out because overuse clearly had no influence on headache pattern.

Patients in the last two groups (B and C) were considered “non-responders.”

The demographic, clinical, and psychological characteristics considered as possible predictors of the above outcomes are reported in Tables 1, 2.

**Table 1:** Demographic and clinical characteristics of the three groups at baseline.

	**Group A**	**Group B**	**Group C**		
	***N* = 118**	***N* = 19**	***N* = 29**		
	**mean ± SD**	**mean ± SD**	**mean ± SD**	***+p***	^*^p
Age	44.7 ± 9.7	41.3 ± 10.7	46.9 ± 10.2	0.17	0.27
Gender (Female) *n* (%)	92 (78%)	17 (89%)	23 (79%)	0.25	0.88
Age at onset	14.7± 8.5	11.7± 7.6	14.2 ± 7.4	0.18	0.81
Drug doses per month	42.4 ± 27.2	53.1 ± 34.6	55.1 ± 65.8	0.17	0.11
Days with intake per month	23.5 ± 5.5	26.3 ± 4.2	23.7 ± 5.5	0.035	0.83
Days with headache per month	25.7 ± 4.5	27.6 ± 3.4	27.0 ± 4.4	0.08	0.16
Duration of overuse (months)	52.5 ± 59.0	80.5 ± 113.2	74.1 ± 71.4	0.10	0.10
Duration of chronic headache (months)	62.5 ± 69.8	113.3 ± 130.0	94.7 ± 81.9	0.012	0.033
Number of previous detoxifications	0.7 ± 1.2	0.7 ± 1.5	1.2 ± 2.3	0.81	0.08
**Preventive therapy upon discharge *n* (%)**				0.93	0.79
None	1 (1)	0 (0)	0 (0)		
B-blockers	10 (9)	1 (5)	1 (3)		
Antiepileptic drugs	29 (24)	6 (32)	7 (24)		
Calcium antagonists	13 (11)	1 (5)	3 (10)		
Tricyclics	38 (32)	7 (37)	8 (28)		
Polytherapies	27 (23)	4 (21)	10 (35)		
**Type of medication overused *n* (%)**				0.41	0.50
Ergotamines	0 (0)	0 (0)	0 (0)		
Triptans	18 (15)	1 (5)	3 (10)		
Opioids	0 (0)	0 (0)	0 (0)		
Analgesics	37 (31)	5 (26)	6 (21)		
Combination of analgesics	11 (10)	1 (5)	3 (10)		
Polyabusers	52 (44)	12 (64)	17 (59)		

*Group A: responders; Group B: subjects who failed to stop overuse; Group C: patients who stopped overuse but maintained a chronic pattern of headache*.

^+^*Comparisons between Group A and Group B*;

* *Comparisons between Group A and Group C*.

**Table 2:** Psychological characteristics of the three groups.

	**Group A**	**Group B**	**Group C**		
	***N* = 118**	***N* = 19**	***N* = 29**		
	**mean ± SD**	**mean ± SD**	**mean ± SD**	***+p***	^*^p
**SCID-I *n* (%)**					
Anxiety	70 (59)	10 (53)	22 (76)	0.58	0.10
Somatoform disorder	25 (21)	8 (44)	6 (21)	0.034	0.75
Eating disorder	0 (0)	0 (0)	0 (0)	0.99	0.99
Panic disorder	9 (8)	4 (21)	5 (17)	0.06	0.11
Dysthymia	14 (12)	2 (11)	6 (21)	0.87	0.21
Bipolar disorder	0 (0)	0 (0)	0 (0)		
Major depression (current)	17 (14)	3 (16)	10 (35)	0.87	0.012
Major depression (history)	38 (32)	8 (42)	17 (59)	0.40	0.008
DOC	7 (6)	3 (15)	5 (17)	0.70	0.62
Phobias	31 (26)	5 (26)	5 (17)	0.99	0.31
**TOTAL PSYCHIATRIC DISORDERS *n* (%)**					
4 or more	8 (7)	4 (21)	5 (17)	0.04	0.08
3	15 (13)	2 (11)	3 (10)	0.79	0.73
2	34 (28)	4 (21)	11 (39)	0.48	0.34
1	28 (24)	6 (32)	7 (24)	0.46	0.96
0	33 (28)	3 (15)	3 (10)	0.26	0.048
**SELF-REPORT QUESTIONNAIRES**					
HADS Depression	6.5 ± 4.1	6.7 ± 4.7	9.4 ± 4.8	0.68	0.001
HADS Anxiety	7.6 ± 3.7	7.5 ± 5.0	9.4 ± 4.8	0.96	0.027
WHOQoL	26.8 ± 4.5	24.5 ± 6.2	23.2 ± 5.7	0.08	0.001
MIDAS	71.0 ± 50.4	114.8 ± 50.5	96.1 ± 59.0	0.015	0.05
**CHILDHOOD TRAUMA QUESTIONNAIRE**					
Total traumatic experiences	1.2 ± 1.3	2.0 ± 1.4	0.9 ± 0.9	0.03	0.44
Emotional traumas	0.8 ± 1.0	1.5 ± 1.4	0.5 ± 0.5	0.018	0.22
Physical traumas	0.4 ± 0.7	0.5 ± 0.7	0.5 ± 0.7	0.60	0.60
Sexual abuse *n* (%)	9 (8)	0 (0)	2 (7)	0.21	0.89
**STRESSFUL LIFE-EVENTS QUESTIONNAIRE**					
Total number of events	15.7 ± 11.2	16.3 ± 9.0	20.8 ± 11.3	0.81	0.028
Mild events	1.0 ± 1.4	0.5 ± 1.0	0.6 ± 1.0	0.38	0.39
Moderate events	1.2 ± 1.4	1.2 ± 1.6	1.2 ± 1.2	0.98	0.94
Important events	2.4 ± 2.2	2.8 ± 1.8	2.7 ± 2.4	0.44	0.55
Serious events	1.0 ± 1.3	0.8 ± 1.0	1.4 ± 1.9	0.61	0.12
Very serious events	0.3 ± 1.0	0.4 ± 1.0	0.8 ± 1.1	0.66	0.001

*Group A: responders; Group B: subjects who failed to stop overuse; Group C: patients who stopped overuse but maintained a chronic pattern of headache*.

^+^*Comparisons between Group A and Group B*;

* *Comparisons between Group A and Group C. SCID-I: Structured Clinical Interview for DSM-IV Disorders; DOC: Obsessive-Compulsive Disorder; HADS: Hospital, Anxiety Depression Scale; WHOQoL: World Health Organization Quality of Life-BREF; MIDAS: Migraine Disability Assessment*.

### Statistical Analysis

Data are presented as means ± SD for continuous data and as n/% for frequency data. The differences between responders (Group A) and non-responders (Groups B and C) to the detoxification program were examined with χ^2^ tests for categorical variables and one-way analysis of variance (ANOVA) for quantitative variables. These analyses were carried out by comparing paired groups (i.e., Group A vs. Group B and Group A vs. Group C). To establish the treatment failure at 2 months, as described above, we applied univariate and multivariate logistic regression (enter method). Variables with significant differences between groups in the ANOVAs were included in the univariate model. Variables with statistical significance at the level of *p* ≤ 0.05 in the univariate analysis were then included in the multivariate models. An alpha of 0.05 was used for all statistical tests. All analyses were conducted using SPSS.

## Result

### Patient Population

**Figure 1:**
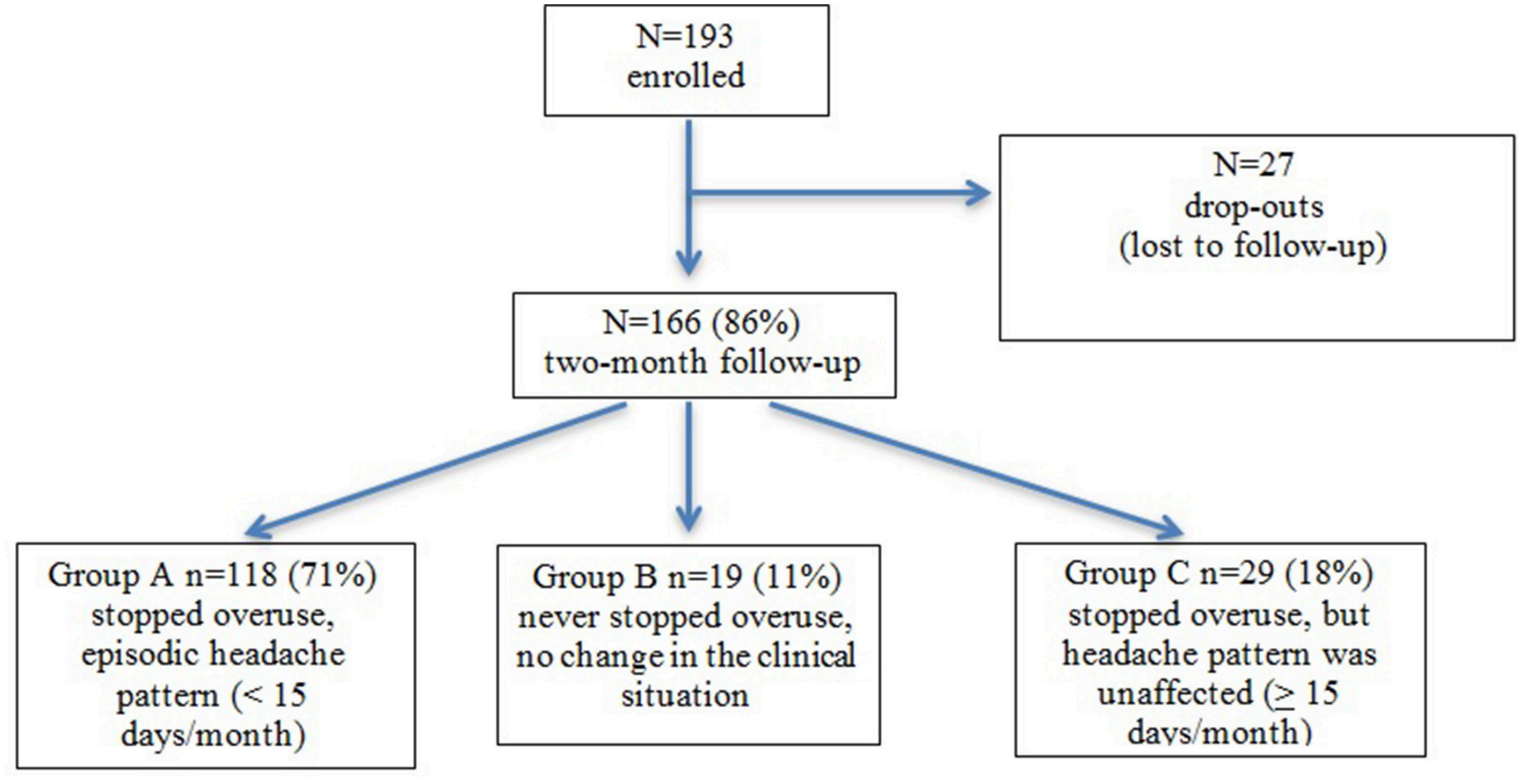
Diagram of the study and clinical pattern at the 2-month follow-up.

As represented in Figure 1, we recruited 193 patients for this study, 166 completed the follow-up at 2 months (80% females; mean age 44.7; age range 21–66). Of these, 118 patients (71%) (78% females, mean age: 44.7; age range 22–66) stopped drug overuse following detoxification and their headache reverted to an episodic form (Group A), 19 patients (11%) (89% females; mean age 41.3; age range 24–65) failed to cease overuse (Group B), and 29 (18%) (79% females; mean age 46.9; age range 21–64) stopped overuse, but their headache kept a chronic pattern (Group C) (Figure 1).

### Comparison Between Group A and Group B

For what concerns demographic and clinical features between these 2 groups, an higher number of days with drugs intake per month [*F*_(1, 135)_ = 4.53, *p* = 0.035] and a longer duration of headache chronicity [*F*_(1, 134)_ = 6.49, *p* = 0.012] resulted in Group B when compared to Group A (Table 1).

In respect of psychological variables (Table 2) assessed via SCID-I, resulted a significant association between group and somatoform disorders, with a higher prevalence of this disorder in Group B (χ^2^ = 4.50; *d.f*. = 1; *p* = 0.034). Moreover, we found a significant association between groups and psychiatric disorders (χ^2^ = 4.17; *d.f*. = 1; *p* = 0.04), with a larger number of persons affected by at least 4 conditions according to SCID-I in Group B than in Group A. Furthermore, Group B was characterized by higher MIDAS scores [*F*_(1, 88)_ = 6.11, *p* = 0.015]. As for traumatic experiences and stressful events, Group B experienced a higher number of childhood traumatic events [*F*_(1, 90)_ = 4.69, *p* = 0.03], in particular of the emotional type [*F*_(1, 89)_ = 5.84, *p* = 0.018].

No other significant differences for what concerns the clinical and psychological variables resulted between Group A and Group B.

#### Predictors of Failure to Cease Overuse

As the results of the univariate analysis (Table 3), the factors related to failure to stop overuse were: higher numbers of days with drug intake and longer duration of headache chronicity, presence of somatoform disorders and presence of at least four psychiatric disorders detected by SCID-I, higher MIDAS scores, and a higher number of traumatic experiences of emotional nature during childhood.

**Table 3:** Model fit of logistic regression equations to predict persistence of overuse at 2 months after detoxification.

	**Univariate OR**	**95% CI**	***p*****-value**	**Multivariate OR**	**95% CI**	***p*****-value**
Days with intake per month	**1.110**	**1.005–1.226**	**0.040**	–	–	0.65
Duration of chronic headache (months)	**1.006**	**1.001**–**1.010**	**0.022**	–	–	0.21
**SCID-I**						
Somatoform disorders (yes vs no)	**2.944**	**1.052**–**8.242**	**0.040**	–	–	0.99
4 or more psychiatric disorders	**3.667**	**0.984**–**13.670**	**0.049**	–	–	0.27
**SELF-REPORT QUESTIONNAIRE**						
MIDAS	**1.016**	**1.002**–**1.030**	**0.024**	–	–	0.31
**CHILDHOOD TRAUMA QUESTIONNAIRE**						
Total traumatic experiences	1.498	1.019–2.202	0.051	–	–	–
Emotional traumas	**1.749**	**1.073**–**2.849**	**0.025**	**11.096**	**1.152**–**106.836**	**0.037**

*Significant OR are in bold. SCID-I: Structured Clinical Interview for DSM-IV Disorders; MIDAS: Migraine Disability Assessment*.

Of the above factors, the only one that survived in the multivariate analysis as predictor of failure to cease overuse was having experienced a higher number of traumatic experiences of the emotional type during childhood. This logistic regression model was statistically significant, χ^2^(6) = 13.77, *p* = 0.032 and it explained 53.0% (Nagelkerke R^2^) of the variance of overuse persistence after 2 months from detoxification. Prediction success overall was 90.9%.

### Comparison Between Group A and Group C

Analysis of demographic and clinical features (Table 1) showed a longer duration of headache chronicity [*F*_(1, 144)_ = 4.61, *p* = 0.033] in Group C.

As regards psychological variables (Table 2), a significant association resulted between group and major depression, both in case of current (χ^2^ = 6.26; *d.f*. = 1; *p* = 0.012) or previous history of depression (χ^2^ = 6.94; *d.f*. = 1; *p* = 0.008), with a higher prevalence of the condition in Group C. Moreover, a significant association resulted between group and the number of psychiatric comorbidities (χ^2^ = 3.91; *d.f*. = 1; *p* = 0.048), with a smaller number of persons not affected by psychiatric conditions (SCID-I) in Group A as compared to Group C.

As regards data collected by self-report questionnaires, patients in Group C showed significantly higher scores than subjects in Group A at the MIDAS [*F*_(1, 99)_ = 3.71, *p* = 0.05]. They also has higher depression [*F*_(1, 145)_ = 10.79, *p* = 0.001] and anxiety [*F*_(1, 145)_ = 5.01, *p* = 0.027] scores at HADS subscales as well as poorer quality of life as detected by the WHOQoL [*F*_(1, 144)_ = 11.39, *p* = 0.001]. As regards stressful live events in the last 10 years, Group C experienced a significantly higher number of life events [*F*_(1, 145)_ = 4.90, *p* = 0.028], especially of the serious type [*F*_(1, 145)_ = 11.38, *p* = 0.001], than Group A.

No other significant differences in the clinical and psychological variables resulted between these two groups of patients.

#### Predictors of Persistence of Chronic Headache

According to the univariate analysis (Table 4,) variables associated to chronic headache were the presence of current or previous of major depression, higher HADS scores for depression and for anxiety, lower WHOQoL scores, higher number of stressful events in the last 10 years, especially of those rated as very serious.

**Table 4:** Model fit of logistic regression equations to predict persistence of chronic headache two months after l detoxification in the subjects who stopped overuse.

	**Univariate OR**	**95% CI**	***p*****-value**	**Multivariate OR**	**95% CI**	***p*****-value**
Duration of chronic headache (months)	1.005	0.971–1.010	0.055	–	–	–
**SCID-I**						
Major depression (current) (yes vs. no)	**3.127**	**1.243**–**7.863**	**0.015**	–	–	0.34
Major depression (history) (yes vs. no)	**2.982**	**1.296**–**6.866**	**0.010**	**3.703**	**1.448**–**9.475**	**0.006**
Absence of psychiatric disorders	0.297	0.084–1.049	0.059	–	–	–
**SELF-REPORT QUESTIONNAIRES**						
HADS Depression	**1.165**	**1.057**–**1.285**	**0.002**	–	–	0.28
HADS Anxiety	**1.123**	**1.011**–**1.246**	**0.030**	–	–	0.68
MIDAS	1.009	1.000–1.018	0.062	–	–	–
WHOQoL	**0.879**	**0.811**–**0.953**	**0.002**	–	–	0.36
**STRESSFUL LIFE-EVENTS QUESTIONNAIRE**						
Total number of events	**1.038**	**1.003**–**1.074**	**0.032**	–	–	0.90
Very serious events	**1.999**	**1.262**–**3.166**	**0.003**	**1.679**	**1.011**–**2.786**	**0.045**

*Significant OR are in bold. SCID-I: Structured Clinical Interview for DSM-IV Disorders; HADS: Hospital, Anxiety Depression Scale; MIDAS: Migraine Disability Assessment; WHOQoL: World Health Organization Quality of Life-BREF*.

In the multivariate analysis, the factors that emerged as predictors of persistence of chronic pattern of headache were: presence of history of major depression and a higher number of very serious stressful events in the last 10 years. This model was statistically significant, χ^2^(7) = 25.68, *p* = 0.001 and it was able to explain 25.6% (Nagelkerke R^2^) of the variance in the persistence of chronic pattern of headache 2 months after detoxification. Prediction success overall was 84.2% of cases.

## Discussion

### Overview

The present findings provide additional information on the importance of trauma and stressful psychological experiences in subjects with CM and associated MOH. More specifically our findings suggest that both childhood distress and stressful episodes have an impact on the failure of the detoxification programme. What is even more interesting is that childhood traumas and current stressful events were differently involved in the two possible negative outcomes that we investigated.

### Childhood Traumatic Events

We found that the failure to stop overuse after detoxification was associated to the presence of emotional (but not physical or sexual) traumas during early life. We know from literature that chronic migraineurs experience a higher number of self-reported physical and emotional traumas when compared to subjects with episodic migraine (11–13). Interestingly, these studies suggest that childhood traumas, especially when of the emotional type, have a role in headache frequency and severity independently from psychiatric comorbidities. This in the face of an association among traumas, headache characteristics, anxiety, and depression, as well-argued by Tietjen (42). Such a finding is confirmed also by our data. A strength of this study consists in having taken in consideration also emotional traumas, which represent a less recognized and explored—and blatant—type of early life adversity then sexual and physical abuse (43). Previous studies have demonstrated how emotional traumas may determine more lasting consequences (44) than physical or sexual abuse (45), including earlier age of migraine onset (11).

Unfortunately, we know much less about the role of psychological trauma in early life in predisposing to the development of medication overuse in headache. In other investigation fields, it has been demonstrated that life misfortunes happened in childhood could be risk factors that increase the likelihood to trigger the development of chronic pain (46–49) as well as to develop a variety of psychiatric disorders, including substance abuse and dependence behaviors (50, 51). The question here is whether the persistence of overuse after detoxification observed in Group B can therefore be interpreted as the result of the presence of dependence behaviors. A previous study by Radat et al. (52) suggested a common underlying vulnerability between MOH and dependence. In contrast with this observation, our group found that MOH and drug addicted patients did not differ in those personality aspects that are specifically related to dependence in cross-sectional studies (53, 54). Considering our findings in the present study and how patients differed according to their detoxification outcome, it is possible to hypothesize that the occurrence of childhood traumas creates a terrain that predisposes subjects to overuse acute headache medications and to persist into this behavior even after the detoxification procedure. The engagement in addictive behaviors is usually related to the need of avoid or regulate negative feelings in those individuals with difficulty in emotion regulation (55, 56). Maltreatment during childhood is able indeed to alter the regular development of emotional processing skills (57). In particular, when people have difficulty in emotion regulation, they experience challenges in engaging in goal-directed behavior, in controlling overriding impulses, and in retrieving efficient emotion-regulation strategies (58, 59). Therefore, our hypothesis is that subjects in Group B tend to have more impulsivity (60) and therefore recur more promptly and easily to acute drugs under the urge of an immediate effect in the face of chronic pain. This also noting the fact that child trauma *per se* may contribute to chronicity, as a consequence of a complex interaction of functional, structural, genetics, and epigenetics changes (61). Such a conclusion is consistent with neurobiological development changes in the brain associated to trauma in early life (62). For instance, voxel-based morphometric studies have shown that adults with childhood traumas present decreased thickness of the orbitofrontal cortex (OFC) (63). Interestingly, the chronic consumption of medications has been associated to OFC dysregulation (hypometabolism), which may lead to the inability to control impulses and to inhibit compulsive drug seeking behaviors (64–66). What is more interesting is that, after withdrawal from overused medications, among all dysmetabolic areas being involved also in pain processing, OFC is the only without metabolic changes (67). Along this line of reasoning, the positive outcome of detoxification in patients belonging to Group A is more likely related to the fact that overuse in this group was caused by the plain need to cope with recurrent pain and not sustained by psychological or behavioral issues. However, our results need to be replicated and extended by assessing the association between early life traumas and dependence.

### Recent Stressful Events

By contrast, when considering subjects that stopped overuse but still presented a chronic pattern of headache, our findings describe a different profile. These patients indeed differed from responders because their higher prevalence of depressive history and the higher number of current stressful life events, in particular of those rated as very serious. In line with previous findings (53, 54), the fact that the subjects belonging to this group managed to discontinue overuse suggests that they did not have a strong dependence-related behavior. From one side, headache is commonly associated to depression (68, 69). The monoamine hypothesis proposes that depression is the result of a dysfunction in neurotransmitters (70), which are also involved in pain modulation (71, 72). Hence, the high prevalence of comorbidity between pain and mood disorders might be explained by the same biological link (73). On the other hand, stress is considered as one of the most common trigger factor for headache and a possible cause for headache chronification (70). The question is whether patients persisting in chronicity are exposed to a greater number of stressful life events or whether they are instead less able to tolerate and cope with stress in comparison with the responders. Only few studies explored the existence of stressors in chronic headache patients than in healthy controls (74, 75). However, such differences concern daily stressors—i.e., daily hassles—whereas here we found differences in term of very serious life events, even if in a very subjective way. Stress, indeed, derives from the number and significance of episodes that happen in life or daily hassles and the inability to deal with them (76). Other Authors (77, 78) have highlighted that chronic headache patients tend to judge very negatively life events and are less able to cope with them in comparison to healthy controls. Keeping this in mind, these findings seem to confirm the hypothesis that a greater difficulty in reacting to stress, also in case of coexisting psychopathological conditions (79, 80), might have a fundamental involvement in the persistence of chronicity also after the elimination of acute medication overuse. Future studies should investigate such aspects in regard with the duration of the experience to these stressors and the impact of depression.

### Implications and Limitations

At the present moment, the topic of migraine in terms of pathophysiology and its chronification is very complex and mostly elusive complex and mostly elusive, being the result of structural and functional alterations (81). With respect to those causative aspects that could determine the transformation from episodic to chronic migraine, we certainly feel that psychological and psychosocial components play a critical role. Before us, others have shown a possible overlap among psychiatric comorbidities, medication overuse headache and risk for suicide (82), findings that highlight the importance of further investigating these complex patients in order to treat them early and prevent negative consequences. Therefore, we think that it is important that psychiatric comorbidity of headache (83) is studied also in the context of early traumas and recent life-events.

Some limitations in our study suggest caution in the interpretation of results and call for further *ad hoc* studies. First of all, we considered a short-term period for the outcome of detoxification. Relying on a 2-months period, we are aware that this period prevents the identification of those patients that will relapse into overuse or that, conversely, might experience an improvement at later times. However, we believe that the 2-month time frame was the most conservative modality for detecting the response to detoxification. Furthermore, we see this investigation as a first step providing important preliminary information for devising other longitudinal studies on these subjects. Collecting data from longer follow-up periods, as well as monitoring over time the same patients of the present study, will likely provide more definite conclusions on this topic. Furthermore, we assessed childhood traumas and current stressors without addressing the duration of such exposure and using retrospective self-report questionnaires. We are aware that this methodology may be affected by recall bias, as well as by bias related to the disease, such as the pain experienced during assessment. However, we are convinced that our findings are valuable for understanding this disorder mostly unknown and they lay the ground for future studies aimed at sorting out the causal and temporal relationship between depression and the modality of reaction to stressful life events. Another critical issue in our findings is their limited transferability to the general practice. Indeed, in our population we had a small number of patients in groups B and C (10 and 18%, respectively); however it is worth noting that these proportions are aligned on those of other studies carried out considering 2-months follow-up (24). Further we had a 14% rate of drop-out, which, though in line with similar studies (25), limits the interpretation of our findings and can only be addressed in future multicentric studies on larger CM+MOH populations (25).

## Conclusion

Our results showed the importance of considering childhood traumas and current life events in subjects with CM+MOH, especially in the response to detoxification. Theoretically, our results are essential for understanding the factors that may be involved in the pathophysiology of CM +MOH and may prove useful for further differentiating this complex group of seriously affected patients in different phenotypical and/or endotypical subtypes. Practically, these data provide useful indications as regards the need of optimizing MOH management by means of a preliminary thorough evaluation of the psychological and psychosocial history. We think that patients fitting with the profile corresponding to Groups B and C in our study should be treated by clinicians with particular attention due to high risk of negative outcome. In particular, by introducing in the therapeutic armamentarium psychotherapeutic approaches, including emotional-focused therapies, have been shown as promising avenues of treatment (84).

## Data Availability

All datasets generated for this study are included in the manuscript and/or the supplementary files.

## Ethics Statement

This study was carried out in accordance with the recommendation and the guidelines of the Ethic Committee of San Raffaele Scientific Institute with written informed consent from all subjects. All subjects gave written informed consent in accordance with Declaration of Helsinki. The protocol was approved by the Ethic Committee of San Raffaele Scientific Institute.

## Author Contributions

SB wrote the first draft. CT and FG performed revisions. All authors contributed to the planning and development of the study, supervised by CT. All authors read and approved the final manuscript.

### Conflict of Interest Statement

The authors declare that the research was conducted in the absence of any commercial or financial relationships that could be construed as a potential conflict of interest. The reviewer LG declared a shared affiliation, though no other collaboration, with one of the authors CT to the handling Editor.
